# Assessing the use of antibiotics in pediatric patients hospitalized for varicella

**DOI:** 10.1186/s13052-022-01393-5

**Published:** 2022-12-12

**Authors:** Elena Bozzola, Silvio Marchesani, Andrea Ficari, Carla Brusco, Giulia Spina, Maria Rosaria Marchili, Stefano Guolo

**Affiliations:** 1 Pediatric Unit, Bambino Gesù Children’s Hospital IRCCS, Rome, Italy; 2 Sanitary Direction Unit, Bambino Gesù Children’s Hospital IRCCS, Rome, Italy

**Keywords:** Varicella, Children, Antibiotic, Cost, Hospitalization, Antimicrobial resistance

## Abstract

**Background:**

Varicella is considered a mild and self-limiting disease, but, in some cases, it may complicate and require hospitalization. Antibiotics are not the first line therapy but in some cases are prescribed either for the management of varicella-related complications or as a preventive strategy. Aim of this study is to analyze the rate and the patterns of antibiotics used in pediatric patients hospitalized for varicella as well as the relative costs in order to increase insights in antibiotic use in varicella.

**Methods:**

Patients less than 18 years hospitalized for varicella at the Bambino Gesù Children’s IRCCS Hospital in Rome, Italy, from the 1^st^ of November 2005 to the 1^st^ of November 2021 entered the study. Retrospective data were collected from the hospital's database electronic medical records. The rate, the patterns and the costs of antibiotics used were considered.

**Results:**

According to the inclusion criteria, we enrolled 810 patients, with a median age of 2.4 years. Out of them, 345 patients (42.6%) underwent antibiotic therapy, of which 307 for a complication (90.0%) and the other 10.0%, antibiotic for the fear of developing complications. The cost for varicella hospitalizations was EUR 2,928,749 (median cost EUR 2689). As for antibiotic therapy, it represented the 5.9% of the total cost (EUR 174,527), with a median cost of EUR 198.8. The cost in patients who underwent antibiotic therapy was significantly higher than in those who did not (*p*-value < 0.0001), as well as the hospitalization length (*p*-value < 0.0001). The most commonly prescribed antibiotics were Amoxicillin-clavulanate and Ceftriaxone, which represented the 36.0% and 25.0% of all antibiotic prescription, respectively.

Antibiotics may negatively affect the economic cost of hospitalization and the prescription is not always in accordance to guidelines, with potential important repercussions on the development of antimicrobial resistance. Actually, resistance to antibiotics is considered a major risk to the future health of the world population as it may lead to longer hospital stay, increased risk of mortality, health care costs and treatment failures.

**Conclusion:**

Strategies to reduce economical cost, hospitalization length and antimicrobial resistance include ensuring appropriate prescription and administration of empiric antibiotics as well as reducing the circulation of preventable infectious diseases through immunization.

**Supplementary Information:**

The online version contains supplementary material available at 10.1186/s13052-022-01393-5.

## Background

Varicella is considered a benign and self-limiting disease, but in a minority of cases, it may lead to complications such as pneumonia, encephalitis, skin superinfections and invasive bacterial infections [1–3]. Therapy in primary care is generally only symptomatic but for individuals who present with, or are at high risk for complications, intravenous or oral administration of antiherpetic agents, such as acyclovir, can be used [4, 5]. Antibiotics aren’t the first line treatment of primary varicella infections, but may be administered to treat bacterial complications such as bacterial superinfection of skin lesions, bacterial pneumonia, sepsis and other bacterial infections thus increasing the hospitalization cost [6–8]. Varicella vaccine has proven to be effective in reducing morbidity and mortality and risk of hospitalization [9–11]. Universal varicella vaccination is currently recommended in 12 European countries and mandatory only in Italy, Hungary and Latvia [12]. The impact of vaccination not only guarantees a protection from the viral disease, but is also associated with reduction in the use of antibiotics, in the costs for varicella management and in the spread of antimicrobial resistance [13–15].

In fact, even if in most cases, the disease is mild, the socio-economic burden is of note. For example, the estimated annual costs of varicella ranges from almost €42 million in Poland to €188 million in Germany, considering either direct and indirect costs [16, 17]. In Italy as well, varicella direct hospitalization costs may be significant, ranging from EUR 558.44 to EUR 42,608.00 [6]. Another global concern is linked to the extensive use of antibiotics and the rise of antimicrobial resistance. Even if many countries, including Italy, promoted multiple awareness campaigns, the prescription of antibiotics for infections with a probable viral origin remains common. In line with this note, a recent study referred that antibiotics are prescribed either for the management of varicella-related complications or as a preventive strategy in absence of a microbiological confirmation [8, 18].

The aim of this study is to analyze the rate and the patterns of antibiotics used in pediatric patients hospitalized for varicella as well as the relative costs in order to increase insights in antibiotic use in varicella. Considering the on-going challenges with antibiotic resistance globally, our report may also support the potential advantage of immunization to prevent varicella complications.

## Methods

### Study design

Patients aged 0–18 years hospitalized for varicella at the Bambino Gesù Children’s IRCCS Hospital in Rome, Italy, from the 1^st^ of November 2005 to the 1^st^ of November 2021 entered the study. Retrospective data were collected from the hospital's database electronic medical records.

The enrolled patients had a clinical diagnosis of varicella, based on the characteristic skin rash. Just in case of a doubt, a polymerase chain reaction (PCR) for varicella virus zoster had been performed. Immunization schedule was reviewed in order to verify whether one or two doses of varicella vaccine had been previously administered.

To be eligible to enter the study, patients just required to be hospitalized in the study period, regardless the reason for hospital admission.

According to literature, a varicella complication may be defined as an unfavorable evolution of the disease, which can become worse in its severity, show a higher number of signs, symptoms or new pathological changes or which become widespread throughout the body, or affect other organ systems. Varicella complications include hematological, neurological, pulmonary, upper respiratory, skin, hepatic, gastrointestinal, urinary, bone complications and sepsis or bacterial superinfection [2, 19]. In case of a suspected sovra-infection, whether the etiological identification is considered of note for the therapeutic approach, blood culture as well as PCR detection of germs by blood sample or swamps are prescribed.

At first, an economic analysis of the hospitalization costs, including hospital accommodation and management, such as procedures and medical and surgical treatment had been performed. Then, patients were split into two groups depending on the treatment during hospitalization. In the first group, patients underwent antibiotic therapy during hospitalization (ABX +) while in the second they did not consume antibiotic therapy (ABX-). Antibiotics use, defined as type, dose, and duration of use, was obtained through patient medical reports.

Varicella-related clinical complications were described and classified by type. Furthermore, we considered four subgroups in ABX + group:

- Subgroup A: patients with a confirmed bacterial infection (clinically diagnosed and microbiologically confirmed)
- Subgroup B: patients with clinically evidence for a bacterial infection
- Subgroup C: frail patients (for age, therapy or comorbidities)
- Subgroup D: patients without an evidence for requiring antibiotics.

Direct medical costs were extracted from the Lazio Regional Health Service Tariffs, referring to the specific codes and expressed in Euro coin.

### Statistical analysis

Statistical analysis was performed to compare and to correlate data. The comparison study between two different groups included the use of Student t-test (two-sided) for parametric distribution or Mann–Whitney test for nonparametric distribution. To compare proportions or categorical outcomes chi-squared test or Fisher’s exact test (when appropriate) were performed. Data considered with statistical significance were those with *p*-value less than 0.05. The statistical analysis and the graphic processing of the data were carried out using the GraphPad Prism software, version 5 for Machintosh (GraphPad Software, Inc).

## Results

According to the inclusion criteria, we enrolled 810 patients, 452 males (55.8%) and 358 females (44.2%), with a median age of 2.4 years (range from 18 days to 17 years). Out of them, 578 patients (71.3%) experienced complications. One death has been reported in the study period.

All the patients did receive neither one nor two doses of varicella vaccination. General characteristics of the study cohort are listed in Table 1.

**Table 1:** General characteristics of the study cohort

**Parameters**	**Value**
Number of patients	810
Median age (years)	2.4
Gender (M/F)%	55.8%/44.2%
Underlying diseases %	18.6%
Mortality %	0.001%
Complications %	71.3%

The median hospital stay was 5 days (range from 1 to 76 days). The median hospitalization cost was EUR 2689 (ranging from EUR 537 to EUR 40,871).

The overall cost for varicella hospitalizations was EUR 2,928,749.

As for antibiotic therapy, it represented the 5.9% of the total cost (EUR 174,527), with a median cost of EUR 198.8.

### *Patients who underwent antibiotic therapy (ABX* +*)*

In our cohort, 345 patients (42.6%) underwent antibiotic therapy (192 males and 153 females, respectively 55.6% and 44.4%, median age 2.9 years). The median hospital stay was 6 days (ranging from 1 to 76 days). Out of them, 307 patients experienced complications (90.0%). Skin and respiratory complications were the most frequently identified among the case series (34.2% and 28.4% respectively). Furthermore, we classified the patients according to the indication of antibiotic therapy. In just 22.6% (subgroup A), a co-infection has been microbiologically identified. As for other 198 patients (57.4%), we observed a clinically evidence for bacterial infection (subgroup B). Finally, 38 patients (11.1%) were classified as frail patients for comorbidities or for age (subgroup C) and 31 patients (8.9%) received antibiotic therapy without a specific reason (subgroup D).

The median cost of hospitalizations was EUR 3227 (from EUR 537 to EUR 40,871). As for the other 10.0%, antibiotic therapy was likely prescribed for the fear of developing complications. The most commonly prescribed antibiotics were Amoxicillin-clavulanate and Ceftriaxone, which represented the 36.0% and 25.0% of all antibiotic prescription, respectively. The overall direct cost for the hospitalization in this cohort of patients was EUR 1,481,046. Of note, the cost for antibiotics therapy represented the 11.8% of the total cost (EUR 174,527). Details on therapy are reported as supplementary materials. (Supplementary Tables 1 and 2).

For 123 patients (35.6%) more than one antibiotic was used during hospitalization.

**Fig. 1:**
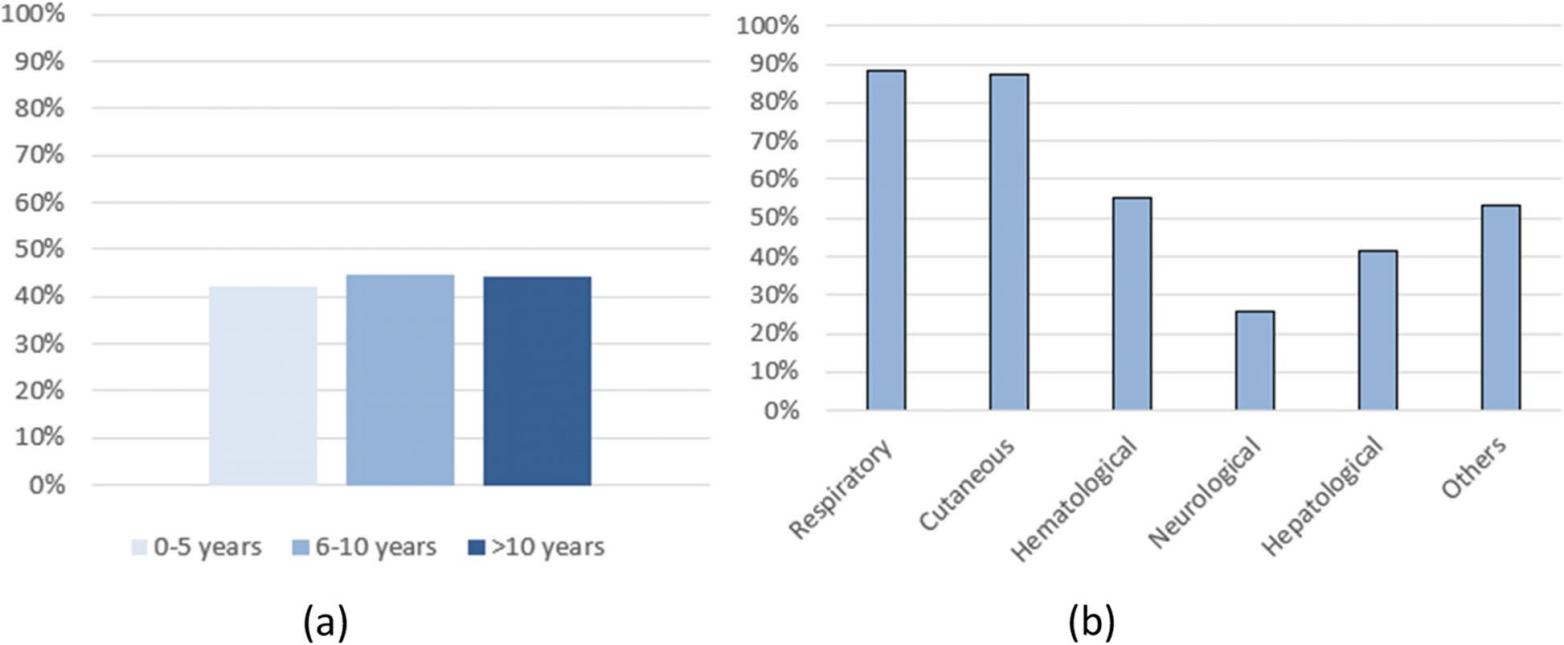
**a** distribution of antibiotic use by age; **b** distribution of antibiotic use by complication

Figure 1 shows the distribution of antibiotic use by age and by complications.

### Patients who did not undergo antibiotic therapy (ABX-)

Out of the cohort, 465 patients (57.4%) did not received antibiotics during the hospitalization (260 males, 55.9% and 205 females, 44.1%, median age 1.3 years). The median hospital stay was 5 days (from 1 to 59 days). In 58.2% of case, they experienced a complication, mainly a neurological (23.2%) and hematological (15.9%) one.

The median cost for hospitalization in this cohort was EUR 2686 (ranging from EUR 537 to EUR 31,729).

### *Comparison between ABX* + *and ABX- groups*

The total cost in the ABX + group was significantly higher compared to the ABX- group (EUR 4356 vs. EUR 3181, *p*-value < 0.0001) with a longer hospital stay (8.1 days vs. 5.9 days, *p*-value < 0.0001). Compared to the ABX-group, patients in the ABX + cohort were significantly older (3.7 years vs. 3 years, *p*-value 0.0062). Data are summarized in Table 2. Complications were more frequent in the ABX + group compared to ABX-group (90.0% vs 58.2%, *p*-value < 0.0001). Respiratory and skin complications were significantly more frequently identified in ABX + group than in ABX- one (*p* < 0.0001). On the contrary, neurological and hematological complications were more likely to be detached in ABX- group than in ABX + (p < 0.0001). Table 3 summarizes the results.

**Table 2:** Comparison between ABX + and ABX- group

	**ABX + **	**ABX-**	***p*-value**
Median age (range) in years	2.9 (0–17)	1.3 (0–17)	0.0062*
Median hospital stay (range) in days	6 (1–76)	5 (1–59)	< 0.0001*
Number of patients who experienced complications (%)	307 (90)	271 (58.2)	< 0.0001*
Median hospitalization cost (range) in EUR	3227 (537–40,871)	2686 (537–31,729)	< 0.0001*
Number of patients with underlying diseases (%)	67 (19.4)	83 (17.8)	0.297

^*^Data with statistical significance. The comparison study between two different groups included the use Mann–Whitney test (nonparametric distribution). To compare proportions chi-squared test were performed

**Table 3:** Main complications identified in ABX + group and ABX- group

**Identified complication**	**Number of patients (%)** **ABX + group**	**Number of patients (%)** **ABX- group**	***p*-value**
**Cutaneous**	118 (34.2%)	17 (3.6%)	< 0.0001*
**Respiratory**	98 (28.4%)	13 (2.8%)	< 0.0001*
**Hematological**	91 (26.4%)	74 (15.9%)	0.0003*
**Neurological**	37 (10.7%)	108 (23.2%)	< 0.0001*
**Hepatological**	20 (5.8%)	28 (6.0%)	0.89
**Others**	63 (18.2%)	53 (11.4%)	0.005*

^*^Data with statistical significance. To compare proportions or categorical outcomes chi-squared test or Fisher’s exact test (when appropriate) were performed

## Discussion

As for the primary aim of this study to describe varicella management in terms of use of antibiotics, with or without complication, 71.3% of the sample size experienced a complication. Out of them, 42.6% underwent an antibiotic treatment.

Just a minority of patients who received an antibiotic treatment (22.6%) had a microbiologically identification of a co-infection. Nevertheless, in many cases microbiological tests had not been prescribed, such as in case of skin superinfection, in which the etiology is very likely to be bacterial. In other patients, blood culture as well as bacterial identification was negative. Nevertheless, patients underwent antibiotic therapy since hospital admission, as therapy should not be delayed while waiting for confirmatory tests, especially among immunosuppressed patients. Suspicion of secondary bacterial infection justify an empirical antibiotic therapy until the results of culture studies, mainly in at high-risk patients, including immunodepressed ones [20].

The most commonly prescribed antibiotics were Amoxicillin-clavulanate and Ceftriaxone which represent the 36%, 25% of all antibiotic prescription, respectively.

The percentage of antibiotics used among varicella patients was higher than the 19.8% reported in Belgium [21]. A main explanation of this is that in our study just hospitalized patients were included, so that the disease course was likely more severe. In line with this note, a multi-country study of the burden of varicella reported that antibiotics were prescribed to nearly 13% of outpatient varicella cases and almost 70% of inpatient cases [8].

Antibiotics are not typically indicated for the management of primary varicella infection, but may be prescribed to treat complications associated with varicella infection such as secondary bacterial skin infections, bacterial pneumonia or other bacterial complications.

Most of the reported antibiotic use in our study is not directly linked to varicella-related complications. Antibiotics were also used in a prophylactic way, to prevent a more severe course of the disease. In our report, we found out that 10% of patients treated with antibiotics did not present a complication but received therapy for the fear of a severe disease.

This is not consistent with international guidelines that strongly advises against the prescription of antibiotics in case of viral infection just for the fear of developing complications. Antibiotics are only indicated in case a concomitant bacterial superinfection is suspected or microbiologically confirmed [22].

These data suggest the use of antibiotics for indications that are not in line with guidelines, with potential important repercussions on the development of antimicrobial resistance. Even if the report does not attempt to evaluate antibiotic resistance directly, it is important to underline that the overuse of antibiotics in the management of varicella may contribute to antimicrobial resistance. Italy is one of the European countries with the higher rate of inappropriate antibiotic prescriptions [23]: antimicrobial drugs were prescribed in 37–61% of hospitalized infants and children [24] and in some cases these prescriptions were unnecessary or inappropriate [25, 26].

Moreover, different studies demonstrated that many children received broad-spectrum antibiotics even if unnecessary, thus increasing the risk of antibiotic resistance appearance [27]. In our case series as well, 123 patients (35.6%) more than one antibiotic was used during hospitalization.

Actually, resistance to antibiotics is recognized as a major risk to the future health of the world population as it may lead to longer hospital stay, increased risk of mortality, health care costs and treatment failures. Strategies to reduce antimicrobial resistance include reducing the circulation of preventable infectious diseases.

Vaccination is a valid and effective tool to prevent infectious diseases, such as varicella, mainly its complications [28]. Our report found out that antibiotics have been mostly prescribed in complicated cases.

Therefore, vaccination may play a role in reducing antibiotics use. Though varicella can also occur among the vaccinated (called breakthrough varicella), such infection is milder, of shorter duration, and more likely to be maculopapular when compared to the unvaccinated, especially after two- dose vaccination. Vaccine implementation program result in a decreased of varicella-related cases, complications and hospitalizations in many Countries including Greece, Spain, Italy and Germany [12, 29, 30].

Finally, previous financial reports have suggested that immunization programs offers economic advantages, largely derived from direct and indirect costs related to varicella prevention [31].

The burden correlated to antibiotic prescription in our study population was of 174,527, representing almost the 6% of total cost for hospitalization. So, antibiotic prescription represents an economic burden for the national health system as confirmed by the results of Agenzia Italiana del Farmaco 2019 report which showed that antibiotic use represents the 3.6% of Italian public health expenditure with an amount of 692,000,000 Euros [23].

This data is even more interesting when considered on a large scale. Scenario analyses comparing no vaccination versus universal vaccination, showed that higher vaccination coverage was also cost saving, reducing annual expenditures for antibiotics in the United States from $82 million to $17 million [13].

## Conclusion

Strategies to reduce either economical cost, hospitalization length and antimicrobial resistance include ensuring appropriate prescription and administration of empiric antibiotics as well as reducing the circulation of preventable infectious diseases through immunization.

## Supplementary Information

**Additional file 1: Supplementary Table 1.** Antibiotics used in varicella hospitalized children. Type of antibiotics used in varicella hospitalized patients are presented as a supplementary table. **Supplementary Table 2.** Annual antibiotic prescription costs in varicella hospitalized children. The annual prescription antibiotic costs of varicella hospitalized children are presented as a supplementary table.

## Data Availability

The Authors confirm that the data supporting the findings of this study are available at the authors ‘office.
