# Supplementary material for: Assessing the use of antibiotics in pediatric patients hospitalized for varicella
Source: Ital J Pediatr. 2022 Dec 12;48:196. doi: 10.1186/s13052-022-01393-5 (PMC9743721; doi:10.1186/s13052-022-01393-5)
Supplement: Supplementary file 1 — Additional file 1: Supplementary Table 1. Antibiotics used in varicella hospitalized children. Type of antibiotics used in varicella hospitalized patients are presented as a supplementary table. Supplementary Table 2. Annual antibiotic prescription costs in varicella hospitalized children. The annual prescription antibiotic costs of varicella hospitalized children are presented as a supplementary table. [file 13052_2022_1393_MOESM1_ESM.docx]

SUPPLEMENTARY MATERIALS

Table 1.

| **Type of antibiotic therapy** | **Number and percentage of patients** |
| --- | --- |
| Amoxicillin-clavulanate | 161 (46,67%) |
| Ceftriaxone | 112 (32,46%) |
| Clarithromycin | 32 (9,27%) |
| Amikacin | 23 (6,67%) |
| Vancomycin | 23 (6,67%) |
| Teicoplanin | 18 (5,21%) |
| Ceftazidime | 15 (4,34%) |
| Gentamicin | 10 (2,89%) |
| Cefixime | 9 (2,6%) |
| Piperacillin-tazobactam | 8 (2,31%) |
| Ampicillin | 6 (1,73%) |
| Meropenem | 6 (1,73%) |
| Amoxicillin | 5 (1,44%) |
| Ciprofloxacin | 4 (1,16%) |
| Linezolid | 4 (1,16%) |
| Trimethoprim-sulfamethoxazole | 4 (1,16%) |
| Levofloxacin | 2 (0,57%) |
| Amphotericin B | 1 (0,29%) |
| Ampicillin-sulbactam | 1(0,29%) |
| Azithromycin | 1(0,29%) |
| Netilmicin | 1(0,29%) |
| Sultamicillin | 1(0,29%) |

Table 2.

| Period time | Number of ABT treated patients | Mean ABT cost (euro) | Total ABT cost (euro) |
| --- | --- | --- | --- |
| November 2005-November 2006 | 51 | 30,99 | 1580 |
| November 2006-November 2007 | 24 | 5,15 | 123,63 |
| November 2007-November 2008 | 36 | 32,54 | 1171,74 |
| November 2008-November 2009 | 38 | 17,82 | 677,41 |
| November 2009-November 2010 | 29 | 85,71 | 2485,60 |
| November 2010-November 2011 | 19 | 17,44 | 331,49 |
| November 2011-November 2012 | 20 | 114,90 | 2292,01 |
| November 2012-November 2013 | 19 | 9,6 | 182,79 |
| November 2013-November 2014 | 29 | 42,67 | 1237,64 |
| November 2014-November 2015 | 25 | 50,74 | 1268,62 |
| November 2015-November 2016 | 30 | 14,35 | 430,79 |
| November 2016-November 2017 | 6 | 46,44 | 278,65 |
| November 2017-November 2018 | 7 | 8,13 | 56,97 |
| November 2018-November 2019 | 9 | 22,42 | 201,862 |
| November 2019-November 2020 | 1 | 50,4 | 50,4 |
| November 2020-November 2021 | 2 | 15,62 | 31,24 |
